# Patterns and Processes in Marine Microeukaryotic Community Biogeography from Xiamen Coastal Waters and Intertidal Sediments, Southeast China

**DOI:** 10.3389/fmicb.2017.01912

**Published:** 2017-10-12

**Authors:** Weidong Chen, Yongbo Pan, Lingyu Yu, Jun Yang, Wenjing Zhang

**Affiliations:** ^1^State Key Laboratory of Marine Environmental Science, Marine Biodiversity and Global Change Research Center, College of Ocean and Earth Sciences, Xiamen University, Xiamen, China; ^2^Aquatic EcoHealth Group, Key Laboratory of Urban Environment and Health, Institute of Urban Environment, Chinese Academy of Sciences, Xiamen, China

**Keywords:** biogeography, community assembly, plankton, benthos, variation partitioning analysis, neutral model, deterministic processes, stochastic processes

## Abstract

Microeukaryotes play key roles in the structure and functioning of marine ecosystems. Little is known about the relative importance of the processes that drive planktonic and benthic microeukaryotic biogeography in subtropical offshore areas. This study compares the microeukaryotic community compositions (MCCs) from offshore waters (*n* = 12) and intertidal sediments (*n* = 12) around Xiamen Island, southern China, using high-throughput sequencing of 18S rDNA. This work further quantifies the relative contributions of spatial and environmental variables on the distribution of marine MCCs (including total, dominant, rare and conditionally rare taxa). Our results showed that planktonic and benthic MCCs were significantly different, and the benthic richness (6627 OTUs) was much higher than that for plankton (4044 OTUs) with the same sequencing effort. Further, we found that benthic MCCs exhibited a significant distance-decay relationship, whereas the planktonic communities did not. After removing two unique sites (N2 and N3), however, 72% variation in planktonic community was explained well by stochastic processes. More importantly, both the environmental and spatial factors played significant roles in influencing the biogeography of total and dominant planktonic and benthic microeukaryotic communities, although their relative effects on these community variations were different. However, a high proportion of unexplained variation in the rare taxa (78.1–97.4%) and conditionally rare taxa (49.0–81.0%) indicated that more complex mechanisms may influence the assembly of the rare subcommunity. These results demonstrate that patterns and processes in marine microeukaryotic community assembly differ among the different habitats (coastal water vs. intertidal sediment) and different communities (total, dominant, rare and conditionally rare microeukaryotes), and provide novel insight on the microeukaryotic biogeography and ecological mechanisms in coastal waters and intertidal sediments at local scale.

## Introduction

Microeukaryotes are found in almost all environments on Earth and cover a wide spectrum of cell sizes, shapes and taxonomic affiliations (Schaechter, 2012; de Vargas et al., 2015). Microorganisms, such as algae, protozoa and fungi play a variety of crucial roles in marine ecosystems from primary producers, predators, decomposers to parasites (Sherr et al., 2007). Microeukaryotic community compositions (MCCs) can vary among marine ecosystems with different environmental conditions (Wang et al., 2014; Massana et al., 2015). Sediment and water column form the two most different and important components of marine ecosystems, each with unique environmental conditions and microbial community structure (Feng et al., 2009; Yang et al., 2016). Such differences may lead to different patterns of microbial biogeography. To date, most studies have separately investigated either planktonic or benthic microbial communities (Gong et al., 2015; Yu et al., 2015). Unfortunately, studies comparing planktonic and benthic MCCs, and the relative influence of environmental/spatial factors on the distributions of planktonic and benthic MCCs in marine ecosystems are still extremely limited (Massana et al., 2015).

Revealing the assembly mechanisms that determine the microbial community composition, and how microbes respond to environmental and spatial change, are major challenges in microbial ecology (Hanson et al., 2012; Liu et al., 2015). The growing efforts to understand these mechanisms mainly focus on two major categories (Sloan et al., 2006; Logares et al., 2013; Liu et al., 2015; Wang et al., 2015a; Yang et al., 2016; Liao et al., 2017). The first one is the deterministic mechanism, which considers that environmental filtering determines the biogeographical pattern of microbes (Gilbert et al., 2012). In general, deterministic mechanism predicts that environmental factors will influence microbial community composition (Dumbrell et al., 2010). A large number of studies have confirmed that microbial community composition might be influenced by a variety of environmental variables including nutrients, salinity, pH, temperature and biotic interactions (e.g., predators) (Fernandez-Leborans et al., 2007; Wang et al., 2014, 2015b; Yu et al., 2015). To fully understand the importance of deterministic processes, the sampling scale should be large enough to incorporate multiscale environmental variables (Yeh et al., 2015). The second category is the stochastic mechanism, which suggests that microbial community assembly is influenced by stochastic processes (e.g., drift). Some studies have quantified the importance of stochastic processes using the neutral model (NM) published by Sloan et al. (2006) since it can correctly explain MCC in diverse environments (Logares et al., 2013; Roguet et al., 2015; Burns et al., 2016). Further, the spatial factors (e.g., distance decay), which are part of the stochastic processes, also cause widespread concern in the study of microbial community assembly. However, some literature suggests that both environmental filtering and stochastic processes simultaneously shape microbial biogeography (Liu et al., 2015; Wang et al., 2015c; Yang et al., 2016), therefore both of these two processes need to be considered when investigating the community assembly of plankton or benthos.

Recently, high-throughput sequencing (HTS) has revealed the biogeography of marine rare biosphere community for bacteria or archaea (Galand et al., 2009; Hugoni et al., 2013), which is essential for understanding overall microbial diversity (Telford et al., 2006). Some HTS studies of microbial diversity have provided insight into distribution patterns, and influencing factors, of both abundant and rare microbes in marine and freshwater systems (Logares et al., 2014; Gong et al., 2015; Liu et al., 2015). For example, Liu et al. (2015) revealed that local environmental factors (e.g., water temperature) significantly affected the distribution of rare bacterial taxa in lakes and reservoirs, whereas spatial factors predominately influenced the distribution of abundant taxa (AT). However, it is still unclear how different environmental and spatial factors affect the distribution of planktonic and benthic abundant/rare microeukaryotic taxa in marine ecosystems (Logares et al., 2014; Lynch and Neufeld, 2015). The abundant taxa (AT) and rare taxa (RT) have significantly different richness and community composition, play different ecological functions, and may have discrepant ecological niches (Liu et al., 2015). Thus, they are likely to have distinct responses to environmental and spatial changes (Pedrós-Alió, 2012; Logares et al., 2013), and we hypothesize that abundant and rare microeukaryotes have different assembly mechanisms between water and sediment habitats.

In this study, we compared the community composition of marine planktonic and benthic microeukaryotes, and their interactions with the environmental and spatial factors. Further, we assessed the relative importance of environmental and spatial factors that structure the distribution of microeukaryotic communities (including total, dominant, rare and conditionally rare taxa) around Xiamen Island, southeast China. We aimed to address the following main questions: (1) How different are the MCCs found in the Xiamen coastal waters and intertidal sandy sediments? (2) How are the distribution of planktonic and benthic MCCs (including total, dominant, rare and conditionally rare taxa) influenced by the environmental and spatial variables? (3) Do stochastic processes explain the community variation of plankton and benthos?

## Materials and Methods

### Study Area, Sampling and Environmental Factors

**FIGURE 1:**
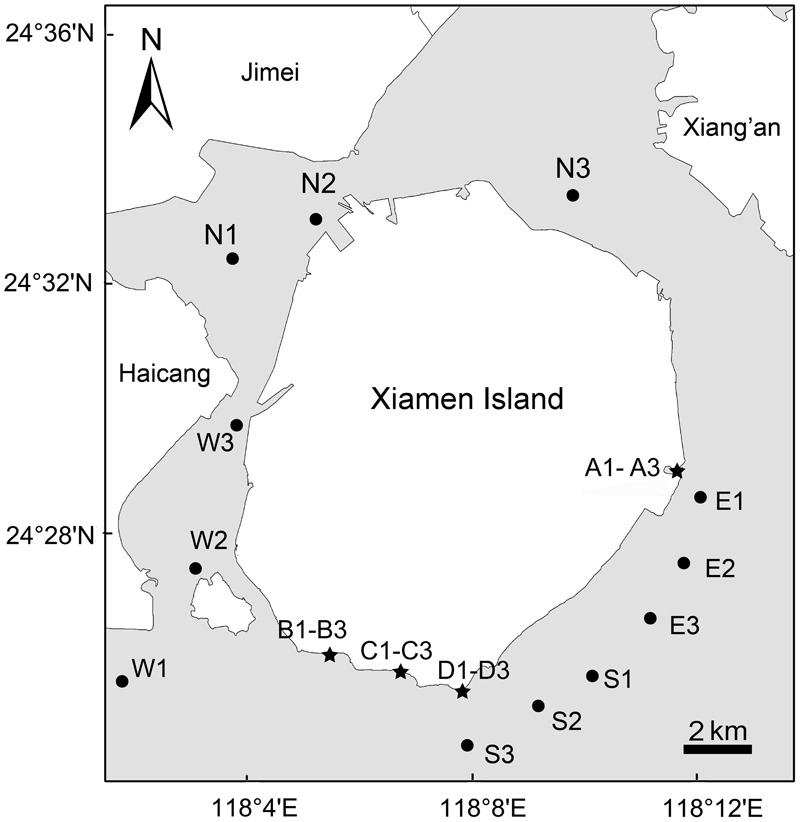
Sketch map of Xiamen island showing the 24 sampling sites in offshore sea water (dots) and sandy beach sediment (stars). A total of 24 samples including 12 surface seawater samples (<0.5 m depth) and 12 surface sediment samples (0–5 cm depth) were collected from Xiamen coastal waters and intertidal sandy beaches in late July 2013 and early September 2014, respectively. The map of Xiamen Island with sampling sites was constructed using ArcGIS 10.1.

Sampling was conducted during two field cruises around Xiamen offshore area (118°01′–118°14′ E, 24°25′–24°34′ N, **Figure 1**). Three biological replicates were selected in water sample stations in the north (N), east (E), south (S), west (W) and sediment stations A, B, C, D to represent of local ecosystem conditions around Xiamen Island (**Figure 1**). The intertidal sandy sediment and coastal water sampling sites were not at the exact same locations, which were ascribed to the intertidal sandy sediment are only distributed in East and South of Xiamen Island. For water samples, the surface (<0.5 m) water samples were transported to the laboratory and processed immediately. For planktonic microeukaryotic community analyses, 800 ml of surface seawater samples were pre-filtered by a 200 μm pore-size sieve to remove debris, large metazoans and grains, and next the water samples with microeukaryotes (smaller than 200 μm) were filtered through 0.22 μm pore polycarbonate membrane (Millipore, Billerica, MA, United States). For sediment samples, the surface (0–5 cm) intertidal sandy sediment samples were transported to the laboratory and processed within 4 h of sampling. The microeukaryotes were mechanically shaken and separated from the sand with sterile seawater over five times, and mixture waters with microeukaryotes were consecutively pre-filtered by a 200 μm pore-size sieve (to remove debris, large metazoans and grains) and filtered/concentrated through 0.22 μm pore polycarbonate membrane (Millipore, Billerica, MA, United States). The membranes were stored at -80°C until DNA extraction. To limit DNA contamination, the sterile seawater was pre-filtered through a nominal 0.22 μm pore-size membrane (Millipore, Billerica, MA, United States). Filtration equipment was rinsed with sterile water before each sample filtering.

At each sampling site, we measured environmental variables (temperature, pH, salinity, turbidity, chlorophyll-*a*, and dissolved oxygen) using a Hydrolab DS5 multiparameter water quality meter (Hach Company, Loveland, CO, United States). Total carbon (TC) and total nitrogen (TN) were analyzed with a TOC/TN-VCPH analyzer (Shimadzu, Tokyo, Japan) and total phosphorus (TP) was determined using spectrophotometry according to established standard methods (Wang et al., 2015b). In addition, a Lachat QC8500 Flow Injection Autoanalyzer (Lachat Instruments, Hach Company, Loveland, CO, United States) was used to determine nitrite and nitrate nitrogen (NO_X_-N), and phosphate phosphorus (PO_4_-P) concentrations.

### DNA Extraction, PCR and Illumina Sequencing

Planktonic and benthic DNAs were extracted by E.Z.N.A. DNA Kit (Omega Bio-Tek Inc., Norcross, GA, United States) and FastDNA SPIN kit (MP Biomedicals, Santa Ana, CA, United States) following the manufacturers’ instructions, respectively. The hyper-variable V9 region of eukaryotic 18S rDNA was amplified using the primers 1380F and 1510R (Amaral-Zettler et al., 2009). Each DNA sample was individually PCR-amplified. The 30 μl PCR mixture contained 15 μL of Phusion Master Mix (New England Biolabs, Beverly, MA, United States), 0.2 μM of forward and reverse primers and 10 ng of the sample DNA. PCR reactions including 1 min initial denaturation at 98°C, followed by 30 cycles of denaturation at 98°C for 10 s, annealing at 50°C for 30 s, extension at 72°C for 60 s. Finally, the amplicons were subjected to 10 min extension at 72°C. Triplicate PCR products for each sample were conducted and purified using GeneJET Gel Extraction Kit (Thermo Scientific, Hudson, NH, United States). Sequencing libraries were generated using NEB Next Ultra DNA Library Prep Kit for Illumina (New England Biolabs, Beverly, MA, United States) according to manufacturer’s instructions, and index codes were added. The library quality was evaluated using the Agilent Bioanalyzer 2100 system (Agilent Technologies, Palo Alto, CA, United States) and Qubit 2.0 Fluorometer (Thermo Fisher Scientific, Waltham, MA, United States). The barcoded amplicons from each sample were mixed in equimolar amounts and were then sequenced using an Illumina Miseq platform (Illumina Inc., San Diego, CA, United States) following a paired-end (2 × 250 bp) approach (Caporaso et al., 2012).

### Bioinformatics

Sequenced paired-end reads were merged with FLASH (Magoč and Salzberg, 2011). Raw data were processed and analyzed using the QIIME v.1.8.0 to remove low-quality reads (Caporaso et al., 2010). Sequences were quality controlled using the following settings: maximum number of consecutive low-quality base = 3; minimum of continuous high-quality base = 75% of total read length; ambiguous bases >0 were removed, last quality score = 3 (Liu et al., 2017). Chimeras were identified and removed using UCHIME before the downstream analyses (Edgar et al., 2011). After that, sequences were clustered into OTUs using UPARSE (Edgar, 2013) with the 97% sequence similarity cut-off. Representative sequences in each OTU were selected and blasted against the SILVA 115 reference database (Quast et al., 2013). Unassigned OTUs (sequence similarity to a reference sequence is <80%) and singletons were discarded prior to further analyses, resulting in a mean of 72048 sequences recovered per sample (range from 33996 to 116717). To minimize biases associated with sequencing coverage and allow for comparison between samples, we randomly selected 33996 sequences from each sample.

### Definition of Abundant and Rare Taxa

We artificially defined thresholds as 0.01% for rare taxa (RT) and 1% for abundant taxa (AT), and classified all OTUs into six categories based on their relative abundance as previously described (Liu et al., 2015; Dai et al., 2016). (1) AT were defined as the OTUs with abundance ≥1% in all samples; (2) RT were defined as the OTUs with abundance <0.01% in all samples; (3) moderate taxa (MT), OTUs with abundance between 0.01 and 1% in all samples; (4) conditionally rare taxa (CRT), with abundance below 1% in all samples and <0.01% in some samples; (5) conditionally abundant taxa (CAT), taxa with abundance ≥0.01% in all samples and ≥1% in some samples but never rare (<0.01%); (6) conditionally rare and abundant taxa (CRAT), OTUs with abundance varying from rare (<0.01%) to abundant (≥1%) (Dai et al., 2016). In this study, we combined AT, CAT, and CRAT as AT to perform further analyses with these three pooled categories taxa (AT, CAT, and CRAT) referred to as ‘dominant taxa’ to avoid confusion. The ecological studies usually consider ‘rarity’ to be a continuous variable, therefore, there is always an effect of arbitrariness when defining a cutoff point for rarity (Gaston, 1994). To reduce the effect of arbitrary definition of dominant and rare OTUs, we performed the Multivariate Cutoff Level Analysis (MultiCoLA) to assess the impact of various abundance or rarity cutoff levels on our resulting data set structure and on the consistency of the further ecological interpretation (Gobet et al., 2010). MultiCoLA is an effective method to systematically explore how large community data sets are affected by different definitions of rarity.

### Analyses of Community Diversity

Rarefaction curve, Venn diagram and alpha diversity indices including OTU richness, ACE (abundance based coverage estimator), Chao 1, Shannon-Wiener, Pielou’s evenness indices and Simpson index of diversity (1-D) were calculated for each sample or entire samples in vegan 2.4-1 with R software (version 3.2.3) (R Core Team, 2015). Good’s coverage was performed in MOTHUR v.1.33.3 software (Schloss et al., 2009). We compared alpha-diversity using one-way ANOVA and Student’s *t*-test.

Bray–Curtis similarity matrix is considered to be one of the most robust similarity coefficients for ecological studies (Kent, 2012) and was applied to our microeukaryotic community dataset. The non-metric multidimensional scaling analysis (NMDS) was employed for detecting possible differences in microeukaryotic planktonic and benthic communities using PRIMER v.7.0 package (PRIMER-E, Plymouth, United Kingdom) (Clarke and Gorley, 2015). Significant difference (*P* < 0.01) between groups was assessed by analysis of similarities (ANOSIM). The analysis of similarities statistic global *R* represents separation degree of between-group and within-group mean rank similarities. *R* = 0 indicates no separation, whereas *R* = 1 indicates complete separation (Clarke and Gorley, 2015).

### Relationships between Community Composition, Environmental Variables, and Geographical Distance

To explore the potential controlling factors for the composition of the microeukaryotic community, we used standard and partial Mantel tests to reveal the correlations between the community similarity and environmental factors. Environmental factors, except pH, were square-root transformed and Euclidean distances between samples were calculated. A geographical distance matrix was calculated based on the longitude and latitude coordinates of each sampling site. Spearman’s rank correlation coefficients were calculated to explore the relationships between the Bray–Curtis similarity of microeukaryotic communities and the geographical distance/environmental factors, and the correlation between the geographical distance and the Euclidean distance of all environmental factors.

We quantified the relative effects of environmental and spatial factors in shaping MCC with variation partitioning analysis (VPA) based on redundancy analysis (RDA), as previously described (Wang et al., 2015c). First, a set of spatial variables were generated through the method of principal coordinates of neighbor matrices (PCNM) analysis (Borcard and Legendre, 2002), based on the longitude and latitude coordinates of sampling sites. Subsequently, variance inflation factors (VIFs) were calculated to check the presence of collinearities among environmental and PCNM variables using the “vegan” package and variables with VIF >10 were removed to avoid the impact of collinearity. In addition, to provide unbiased estimates of the variation partitioning based on RDA, microeukaryotic data were Hellinger-transformed prior to the analyses (Legendre and Gallagher, 2001). Forward selection procedure was used to select environmental and spatial variables (Blanchet et al., 2008). Finally, VPA was performed using the “varpart” function of the package vegan. For the seawater habitat, we artificially removed sites N2 and N3 due to their distinct microeukaryotic plankton communities compositions with 10 other sites based on the results of NMDS analyses, and also performed both Mantel tests and VPA using the 10 other water samples.

### Neutral Community Model for Microeukaryotes

We evaluated the fit of the Sloan neutral community model for MCCs to determine the potential importance of stochastic processes (Sloan et al., 2006, 2007), which considers OTU occurrence frequency in a set of local communities, and their regional relative abundance across the wider metacommunity. The model used here is an adaptation of the neutral theory (Hubbell, 2001) adjusted to be appropriate for large microbial populations. In this model, *Nm* is an estimate of dispersal between communities. The parameter *Nm* determines the correlation between occurrence frequency and regional relative abundance, with *N* describing the metacommunity size and *m* being immigration rate (Sloan et al., 2006). In this study, we separately used the following data sets – seawater, sediment, and all 24 samples. Further, for seawater habitat, we assessed the fit of the NM for all 12 water samples and 10 water samples (without sites N2 and N3), respectively. All computations were performed in R (version 3.2.3) (R Core Team, 2015).

### Accession Number

All raw sequences from this study have been submitted to the National Center for Biotechnology Information (NCBI) Sequence Read Archive (SRA) database under the BioProject number PRJNA342297 and the accession number SRP089752.

## Results

### Comparison of Environmental Factors between Water and Sediment Pore Water

The environmental factors of the studied sites were summarized in Supplementary Table S1. In general, the dynamics of environmental factors from surface waters were more complex than those from intertidal sediment pore waters. The temperature in sediment sites (31.5–33.9°C) was generally higher than water sites (28.1–30.2°C) except N2 and N3 (31.5 and 31.7°C, respectively). Mean values of salinity and pH in the water sites (26.4 psu and 9.35) were higher than sediment sites (25.0 psu and 7.97). However, both TN and TP showed higher mean values in sediment sites (3.07 and 0.112 mg l^-1^) than water sites (0.57 and 0.018 mg l^-1^).

### Comparison of Richness and Diversity between Plankton and Benthos

Benthic communities showed a significantly higher species richness compared to planktonic communities (Supplementary Figures S1–S3), although Shannon-Wiener, Simpson index of diversity and Pielou’s evenness indices did not exhibited significant differences. Rarefaction curves for each sample was seldom saturated (Supplementary Figure S1A). However, the global rarefaction curve indicated that sequencing of ∼0.81 million V9 rDNA sequences from 24 samples was sufficient to approach saturation of microeukaryotic richness (Supplementary Figure S1B). Further, the total number of microeukaryotic OTUs (9003) was roughly equivalent to the number estimated by abundance based richness estimators such as Chao 1 (9005 ± 1.74) and ACE (9026 ± 47) (Supplementary Table S2). Good’s coverage ranged from 96.62 to 98.92% in each sample and the index of all 24 samples combined was 99.99%, respectively (Supplementary Table S2). The rarefaction curves, extrapolated species richness indices (Chao 1 and ACE) and Good’s coverage indices indicated that the majority of the microeukaryotic taxa had been recovered from the studied metacommunity (Supplementary Figure S1 and Table S2).

A total of 9003 microeukaryotic OTUs were identified from 815 904 high-quality reads at 97% identity level for the 24 samples. Further, Supplementary Table S3 summarized the contribution of each microbial taxa category to microeukaryotic community. MultiCoLA showed that when the structure patterns of community data were compared between the truncated and the original matrices, little variation in data structure was observed after removing up to 60% of the rare types, indicating consistent ecological patterns between the two different matrices. In addition, when the increasing amount of rare types was >5%, consistent ecological patterns were also maintained between the truncated and the original matrices (Supplementary Figure S4). These results indicate that our definitions of dominant taxa (47.54%) and RT (1.88%) were reasonable and objective in this study.

The planktonic microeukaryotic metacommunity generated 4044 OTUs, while the benthic microeukaryotic metacommunity generated 6627 OTUs with the same sequencing depth (Supplementary Figure S3). It appeared that the absolute number of OTUs was higher in benthic than planktonic metacommunities. The most abundant OTUs that had mean relative abundance >1% represented 29.88% and 21.39% sequences in 12 water and 12 sediment samples, respectively. All of these OTUs were classified as either Stramenopiles or Animalia. In addition, one Arthropoda OTU (OTU_3) and one Diatomea OTU (OTU_7452) were present in both water and sediment habitats with mean relative abundance ≥1% (Supplementary Table S4).

### Comparison of Microeukaryotic Community Compositions

**FIGURE 2:**
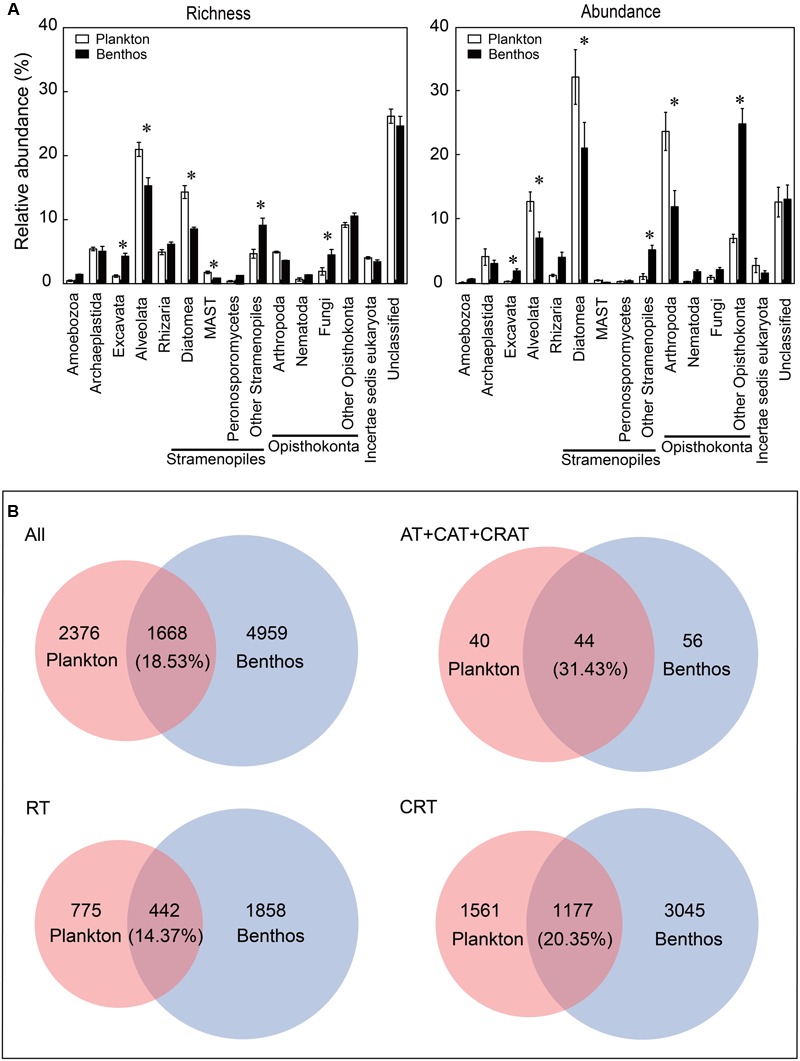
Comparison of microeukaryotic community between planktonic and benthic taxa at supergroup and OTU levels. **(A)** Relative abundance of richness (OTU numbers) and abundance (sequence numbers) of planktonic microeukaryotic taxa compared with benthic microeukaryotic taxa. Unclassified eukaryotes (sequence similarity >80%). Values are means ± SE (*n* = 12); ^∗^*P* <0.05 (Student’s *t*-test). **(B)** Venn diagrams showing the number and percentage of OTUs that are unique and shared between the microeukaryotic plankton and benthos. All, all taxa; AT, abundant taxa; CAT, conditionally abundant taxa; CRAT, conditionally rare and abundant taxa; RT, rare taxa; CRT, conditionally rare taxa.

Overall, the most diverse and abundant OTUs in both water and sediment habitats were assigned to the groups of Opisthokonta (mean relative abundance: 15.64% OTUs and 31.81% sequences for plankton vs. 19.63% OTUs and 40.98% sequences for benthos, respectively) and Stramenopiles (21.13% OTUs and 33.94% sequences for plankton vs. 19.69% OTUs and 27.17% sequences for benthos, respectively) (**Figure 2A**). Some groups such as Alveolata (21.84% OTUs and 12.82% sequences for plankton vs. 14.77% OTUs and 7.11% sequences for benthos, respectively) and Diatomea (14.13% OTUs and 31.88% sequences for plankton vs. 8.37% OTUs and 21.06% sequences for benthos, respectively) exhibited significantly higher abundance and diversity in the water habitat compared with the sediment habitat. However, some groups such as Excavata (1.19% OTUs and 0.33% sequences for plankton vs. 4.28% OTUs and 1.97% sequences for benthos, respectively) exhibited an inverse trend, which were more diverse and abundant in benthic habitat (**Figure 2A**). Further, Venn diagrams indicated that benthic microeukaryotes were more diverse than plankton (including total, dominant taxa, RT, and CRT), since more OTUs were only presented in sediment than in water habitats (**Figure 2B**).

**FIGURE 3:**
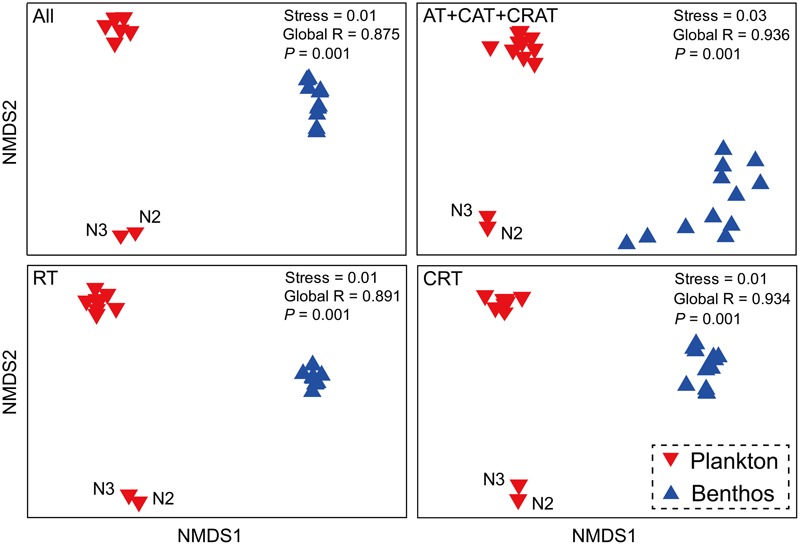
Microeukaryotic community beta-diversity visualized using non-metric multidimensional scaling (NMDS) based on Bray–Curtis similarity. All, all taxa; AT, abundant taxa; CAT, conditionally abundant taxa; CRAT, conditionally rare and abundant taxa; RT, rare taxa; CRT, conditionally rare taxa.

There was a clear difference between planktonic and benthic communities with high global *R*-values in the ANOSIM tests (**Figure 3**). Interestingly, planktonic communities were further separated into two subgroups, the first subgroup comprised sampling sites N2 and N3, while the second contained 10 other sample sites. Further, the total, dominant, rare and conditionally rare communities showed similar patterns in all NMDS plots.

### Relationships between Environmental, Spatial Factors and Microeukaryotic Community

The Mantel tests showed that three physicochemical variables (salinity, TP, and TN) were significantly related to the changes of all of the four categories (total, dominant taxa, RT, and CRT) of benthic communities (**Table 1**). Further, both PO_4_-P and NO_X_ were found to be significant determinants of the total, dominant and CRT subcommunities (*P* <0.05). Temperature was significantly related to the variation of dominant and CRT for the benthic community. However, the Mantel tests for the planktonic communities (including 12 water sites and 10 water sites) showed that no environmental factor was significantly related to the variation of planktonic community composition.

**Table 1:** Mantel tests for the correlations between geographical distance, environmental distance, individual environmental factors, and microeukaryotic community similarity in water and sediment using Spearman’s coefficients.

Factors	Habitat	All taxa		AT+CAT+CRAT		RT		CRT	
		*r*	*P*	*r*	*P*	*r*	*P*	*r*	*P*
Geo_distance	Water (12 sites)	-0.214	0.085	-0.160	0.198	-0.130	0.298	-0.201	0.106
Env_distance	Water (12 sites)	-0.057	0.532	-0.061	0.517	-0.171	0.775	-0.111	0.696
Temperature	Water (12 sites)	-0.150	0.838	-0.239	0.942	-0.259	0.978	-0.232	0.981
Salinity	Water (12 sites)	0.123	0.224	0.071	0.334	0.115	0.239	-0.008	0.403
pH	Water (12 sites)	-0.128	0.675	-0.137	0.712	-0.171	0.754	-0.136	0.716
TN	Water (12 sites)	0.026	0.353	0.033	0.488	-0.001	0.439	-0.127	0.732
TP	Water (12 sites)	-0.050	0.526	0.168	0.172	0.001	0.434	0.077	0.306
Turbidity	Water (12 sites)	-0.057	0.588	0.086	0.238	0.207	0.105	0.193	0.111
Chlorophyll-a	Water (12 sites)	-0.127	0.739	-0.089	0.625	-0.148	0.774	-0.157	0.829
DO	Water (12 sites)	-0.016	0.450	-0.123	0.689	-0.127	0.717	-0.108	0.661
Geo_distance	Sediment (12 sites)	-**0.622**	**0.001**	-**0.400**	**0.001**	-**0.579**	**0.001**	-**0.798**	**0.001**
Env_distance	Sediment (12 sites)	**0.407**	**0.002**	**0.411**	**0.004**	0.191	0.067	**0.413**	**0.007**
Temperature	Sediment (12 sites)	0.342	0.062	**0.374**	**0.009**	0.069	0.243	**0.330**	**0.008**
Salinity	Sediment (12 sites)	**0.675**	**0.001**	**0.636**	**0.001**	**0.384**	**0.014**	**0.659**	**0.001**
pH	Sediment (12 sites)	0.056	0.350	0.052	0.357	-0.106	0.739	0.066	0.252
TN	Sediment (12 sites)	**0.250**	**0.034**	**0.189**	**0.041**	**0.521**	**0.002**	**0.322**	**0.015**
TP	Sediment (12 sites)	**0.308**	**0.009**	**0.292**	**0.043**	**0.337**	**0.015**	**0.295**	**0.031**
TC	Sediment (12 sites)	0.072	0.267	0.068	0.266	0.065	0.645	0.002	0.458
NOX -N	Sediment (12 sites)	**0.254**	**0.046**	**0.282**	**0.023**	0.183	0.091	**0.279**	**0.042**
PO_4_-P	Sediment (12 sites)	**0.358**	**0.004**	**0.443**	**0.002**	0.212	0.064	**0.448**	**0.003**
Geo_distance	Water (10 sites)	0.029	0.850	0.097	0.526	0.162	0.287	-0.008	0.958
Env_distance	Water (10 sites)	0.205	0.166	0.043	0.405	0.090	0.365	0.364	0.053
Temperature	Water (10 sites)	0.134	0.231	0.066	0.393	0.396	0.067	0.310	0.078
Salinity	Water (10 sites)	-0.155	0.766	-0.319	0.957	-0.297	0.93	0.067	0.302
pH	Water (10 sites)	0.131	0.244	0.030	0.401	0.019	0.417	0.206	0.194
TN	Water (10 sites)	-0.096	0.616	-0.238	0.870	-0.232	0.904	0.084	0.271
TP	Water (10 sites)	0.237	0.099	0.171	0.180	0.115	0.234	0.241	0.111
Turbidity	Water (10 sites)	0.112	0.261	0.075	0.312	-0.047	0.572	-0.006	0.447
Chlorophyll-a	Water (10 sites)	0.178	0.156	0.119	0.237	0.242	0.090	0.261	0.081
DO	Water (10 sites)	0.505	0.057	0.440	0.056	0.331	0.084	0.474	0.061

*Geo_distance, pairwise geographical distances between sampling sites; Env_distance, Euclidean distance of all environmental variables between sampling sites; Community similarity was based on the Bray–Curtis similarity. The significances are tested based on 999 permutations, and bold values indicate statistical significance (*P* < 0.05). Water samples with 10 sites indicated removing sites N2 and N3. All, all taxa; AT, abundant taxa; CAT, conditionally abundant taxa; CRAT, conditionally rare and abundant taxa; RT, rare taxa; CRT, conditionally rare taxa. TN, total nitrogen; TP, total phosphorus; DO, dissolved oxygen; TC, total carbon; NO_*X*_-N, nitrate and nitrite nitrogen; PO_*4*_-P, phosphate phosphorus.*

The Bray–Curtis similarity in the total, dominant, rare and conditionally rare benthic communities between any pair of samples significantly decreased with the increasing of geographical distance (*P* < 0.01, **Table 1**), indicating that sites in sandy sediments which were geographically distant had less similar communities – presumably because of dispersal limitation. However, no significant distance-decay relationship was found for planktonic communities (including both of 12 sites and 10 sites, **Table 1**). Further, both planktonic and benthic samples that were closer to each other showed more similar environmental conditions (*P* < 0.01, Supplementary Figure S5). However, benthic sites exhibited a stronger environment-distance relationship than planktonic sites (Spearman’s correlation coefficient 0.378 for planktonic sites and 0.839 for benthic sites, respectively).

The VPA indicated that both environmental and spatial factors play significant roles in structuring microeukaryotic metacommunity in water and sediment habitats (**Table 2**). For total and dominant taxa, the spatial factors exhibited a slightly higher contribution to the community variation than environmental factors, however, a high proportion of unexplained variation was observed in the RT (78.1–97.4%) and CRT (49.0–81.0%) (**Table 2**). For total MCC, the variance explained by purely spatial factors was slightly higher (27.1% vs. 24.2% for plankton and 30.2% vs. 19.1% for benthos, respectively) than that of purely environmental factors in both habitats. For the dominant subcommunity, spatial factors alone also explained higher community variations (28.5% vs. 23.2% for plankton and 33.0% vs. 18.5% for benthos, respectively) than the environmental factors. Interestingly, for the planktonic samples after removing sites N2 and N3, spatial factors exhibited a much higher contribution to the community variations than environmental factors among all of total, dominant taxa, RT, and CRT. Moreover, the contribution of environmental factors to the planktonic MCC variations exhibited a rapid decline among all of total (3.4%), dominant (11.0%), rare (0%), and conditionally rare (4.2%) planktonic taxa. For the benthic microeukaryotic community, the unexplained variation of the RT (78.1%) and CRT (49.0%) was higher than that of the total (46.3%) and dominant (40.4%) communities. Similarity, in the water sites (12 sites and 10 sites, respectively), the unexplained variation of the RT (87.1 and 97.4%, respectively) and CRT (53.3 and 81.0%, respectively) was also higher than that of the total (44.6 and 76.2%, respectively) and dominant (43.2 and 60.3%, respectively) communities.

**Table 2:** Variation partitioning of the microeukaryotic communities among environmental and spatial variables in water and sediment.

Habitat	Effects	All		AT+CAT+CRAT		RT		CRT	
		Adj.*R*^2^	*P*	Adj.*R*^2^	*P*	Adj.*R*^2^	*P*	Adj.*R*^2^	*P*
Water (12 sites)	Environment	0.242	0.144	0.232	0.227	0.032	0.252	0.130	0.105
	Spatial	**0.271**	**0.023**	**0.285**	**0.008**	0.045	0.061	0.104	0.039
	Shared	0.041		0.051		0.052		0.233	
	Residual	0.446		0.432		0.871		0.533	
Sediment (12 sites)	Environment	**0.191**	**0.005**	**0.185**	**0.005**	0.113	0.057	**0.202**	**0.003**
	Spatial	**0.302**	**0.007**	**0.330**	**0.025**	0.092	0.368	**0.248**	**0.001**
	Shared	0.044		0.081		0.014		0.060	
	Residual	0.463		0.404		0.781		0.490	
Water (10 sites)	Environment	0.034	0.063	0.110	0.075	0	0.202	0.042	0.088
	Spatial	**0.174**	**0.033**	**0.317**	**0.013**	0.031	0.176	0.133	0.125
	Shared	0.030		0		0.034		0.015	
	Residual	0.762		0.603		0.974		0.810	

*Environment, community variation explained by pure environmental factors; Spatial, community variation explained by pure spatial factors; Shared, community variation explained by joint effect of environmental and spatial factors. Bold values indicate statistical significance (*P* < 0.05). Negative value of adjusted coefficients of determination (Adjusted *R*^*2*^) was converted to zero. Water (10 sites) indicated removing sites N2 and N3.*

### Fit to the Neutral Model of Community Assembly

**FIGURE 4:**
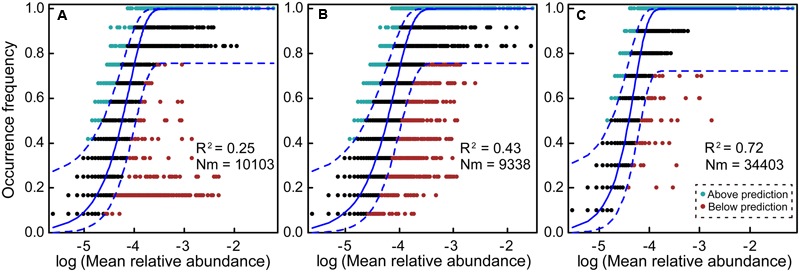
Fit of the neutral model (NM) of community assembly. The predicted occurrence frequencies for plankton and benthos representing microeukaryotic communities from seawater and sediment, respectively. The solid blue lines indicate the best fit to the NM as in Sloan et al. (2006) and dashed blue lines represent 95% confidence intervals around the model prediction. OTUs that occur more or less frequently than predicted by the NM are shown in different colors. *Nm* indicates metacommunity size times immigration, *R*^2^ indicates the fit to the NM. **(A)** 12 plankton communities; **(B)** 12 benthic communities; **(C)** 10 plankton communities (after removing sites N2 and N3).

The NM estimated a low fraction of the variation in the frequency of occurrence of different OTUs in planktonic communities (**Figure 4A**, *R*^2^ = 0.25). The benthic microeukaryotic community showed a larger fraction of the variation in the frequency of occurrence than planktonic microeukaryotic community (**Figure 4B**, *R*^2^ = 0.43), indicating a medium fit to the NM, although the Nm-value was higher for planktonic communities (Nm = 10103) than for benthic communities (Nm = 9338). We also tested the fit to the NM for 10 planktonic communities excluding sites N2 and N3, and found the highest Nm-value (Nm = 34403). Interestingly, this model explained the largest proportion of the variability in occurrence frequency of planktonic community without sites N2 and N3 among the all different data-sets (**Figure 4C**, *R*^2^ = 0.72). Further, we evaluated the fit to neutral community model on all 24 planktonic and benthic communities, and found that the observed data exhibited no fit to the neutral curve (Supplementary Figure S6, *R*^2^ = -0.09, note that negative *R*^2^-values can occur when there is no fit to the model), indicating a strong role for habitat filtering relative to stochastic processes.

## Discussion

### Microeukaryotic Composition and Diversity Patterns in Water and Sediment

Our DNA metabarcoding results validated a well-known observation that planktonic and benthic microeukaryotic communities composition and structure are significantly different (Zinger et al., 2011; Massana et al., 2015; Forster et al., 2016), which was evident in the alpha-diversity (e.g., OTU richness and ACE index), beta-diversity and taxonomic composition (e.g., Diatomea) (**Figures 2, 3** and Supplementary Figures S2, S3). The comparison of MCCs between water and sediment habitats provided additional insight into the diversity and distribution of subtropical marine microeukaryotes.

The microeukaryotic community differences between planktonic and benthic taxa held at both supergroup and OTU levels, although 18.53% OTUs were common to both habitats (**Figure 2**). First, the dominant overlap between benthic and planktonic taxa in our data was related to Alveolata, Stramenopiles (represented mainly by Diatomea) and Opisthokonta (represented mainly by Arthropoda). Although a 200 μm pore-size sieve was used to remove large metazoans, we found that the Opisthokonta was an abundant group, and they were dominated by Arthropoda, which accounted for 23.62% of planktonic sequences and 11.76% of benthic sequences. One explanation for this is that some large-sized organisms can get through the 200-μm pores and/or presence of eggs, spores, or larvae of the large-sized organisms (Liu et al., 2017). Another alternative explanation for this is that our DNA-based molecular approach cannot exclude the potential effect by “free DNA in the water” or “animal debris” passing the 200 micron filter on the perceived microeukaryote communities (Thomsen and Willerslev, 2015; Liu et al., 2017). Both Alveolata and Stramenopiles are dominant benthic microeukaryotic groups, as is also shown by previous studies (Gong et al., 2015; Forster et al., 2016). However, our finding showed that Alveolata was more dominant in water habitat. Further, another study (Mann and Evans, 2007) showed that Diatomea were more often detected in the benthos compared with plankton among phototrophic protists, which is in contrast to our data. This difference might be ascribed to different sampling areas in these various studies. Excavata is a major eukaryotic supergroup, however, only a few studies have focused on it (Hampl et al., 2009) compared to other groups (e.g., Alveolata and Diatomea). Second, our study showed that benthic microbial communities exhibited higher richness than planktonic communities although there was a little overlap between the two communities (**Figure 2B**). Studies in the Gulf of Maine and Long Island Sound in North America also revealed little overlap between genetic signatures of the water and sediment microeukaryote assemblages (Doherty et al., 2010). One explanation for this observation might be that benthic habitats exhibited stronger horizontal and vertical gradients in both physical and chemical characteristics. Environmental heterogeneity is much more pronounced in the benthic than in the planktonic sites, and higher environmental heterogeneity likely promotes the existence of highly specialized organisms, thereby probably driving species-richness patterns (Hortal et al., 2009).

Although further investigations are necessary to characterize the effects of DNA extraction protocols and PCR methods on MCCs, the robust patterns in microeukaryotic community were distinct between coastal waters and intertidal sediments. Recently, a study of amplicon-based characterization of microbial community structure revealed that using different DNA extraction kits did not significantly affect alpha diversity/richness, beta diversity and dominant members of the community of samples (Staley et al., 2015). Therefore, we consider that different DNA extraction kits did not significantly affect the overall biological conclusions drawn in this study. Our NMDS analysis showed that communities from the same habitat cluster together (**Figure 3**), which indicated a distinct composition among planktonic and benthic communities. Further, in the water habitat, the differences between sites N2 and N3 compared to the 10 other water sites have been showed by a previous study (Yu et al., 2015).

### Controlling Factors Shaping the Microeukaryotic Community Compositions

The distinct distribution patterns of MCCs between water and sediment could be attributed to two types of mechanisms: environmental filtering and stochastic processes (Liu et al., 2015; Yang et al., 2016; Liao et al., 2017).

First, environmental factors such as nutrient concentrations can influence MCCs because they are essential for the growth and development of microorganisms, and different microorganisms are adapted to their optimal growth concentrations (Kneip et al., 2007; Wang et al., 2015b). MCCs can be both directly and indirectly affected by nutrient concentrations, which can influence the photosynthesis of autotrophs, and heterotrophic microeukaryotes which can prey on autotrophs (Hecky and Kilham, 1988). In this study, the Mantel tests revealed that benthic MCCs (total, dominant taxa, RT, and CRT) were significantly correlated with salinity, TP and TN in sediments (**Table 1**). These results are consistent with studies of bacterioplanktonic or microeukaryotic communities responses to TP or TN concentrations (Liu et al., 2011; Wang et al., 2015a). Further, salinity is also an important environmental factor affecting the survival and growth of marine microeukaryotes. Changes in salinity directly affect the osmoregulation and metabolism of microorganism, leading to effects on the life activities of microorganism (Decamp et al., 2003). However, our results indicate that there is a clear difference in correlation between plankton and benthos with environmental factors (**Table 1**). Interestingly, no measured environmental variable was significantly associated with planktonic MCC (including 12 water sites and 10 water sites). There are at least two reasons that might possibly explain the observed non-significant relationship between plankton and environmental variables. The first possibility is that plankton communities are extremely dynamic, and snapshot or even regular but low resolution sampling includes a large fraction of noise, which may mask main ecological patterns (Özkan et al., 2014). Second, variation in planktonic communities not explained by environmental (e.g., abiotic) controls might indicate strong biotic interactions (Wang et al., 2015b). In addition, there are some potential limitations that merit further discussion. Some unmeasured environmental variables, such as irradiance and ocean currents, are important in shaping the distributions of MCCs (Yeh et al., 2015). The low statistical power for detecting patterns was perhaps largely due to small sample size in field metacommunity studies, and the sampling scale should be considered when designing sampling schemes (Martiny et al., 2011). That is, the sampling size should be large enough to include most important environmental gradients and dispersal factors.

Second, an increasing body of literature in microbial community ecology indicates that spatial factors are another major process shaping biogeographical patterns besides environmental selection (Vyverman et al., 2007; Cermeno and Falkowski, 2009; Hanson et al., 2012). Our results showed that spatial effects were significant in shaping benthic MCCs (total, dominant taxa, RT, and CRT), whereas marine planktonic MCCs (including 12 water sites and 10 water sites) do not adhere to significant distance-decay relationship (**Table 1**). Recently, Gong et al. (2015) surveyed microeukaryotes of coastal sediment sites in the Yellow Sea and revealed limited dispersal of benthic microeukaryotes, which showed a similar distribution pattern to our results. Comparable and similar results have been noted for benthic foraminifera in deep sea and benthic diatoms in lakes (Telford et al., 2006). However, marine planktonic picoeukaryote such as MAST-4 did not associate with significant distance-decay pattern, but the environmental selection (e.g., temperature) drove MAST-4 distribution (Rodríguez-Martínez et al., 2013). Another study on marine planktonic diatom assemblages also showed that these eukaryotic microbes were not limited by dispersal (Cermeno and Falkowski, 2009). Overall, these results suggest that benthic and planktonic microeukaryotes might have fundamentally different biogeographical patterns or ecological mechanisms. There are large differences in benthos compared to plankton including life environment, food-web and body structure (Schaechter, 2012). In sediments, movements of microeukaryotes are obviously more limited relative to water columns, where planktonic microbes can disperse passively to distant locations because of ocean currents (Yeh et al., 2015). These differences can lead to distinct biogeographical patterns between plankton and benthos at a regional scale.

More importantly, our results show that the rare and conditionally rare benthic subcommunities follow the similar and general biogeographical distribution patterns with dominant taxa, because all of the three subcommunities exhibited significant distance-decay patterns (**Table 1**). This could be because of dispersal limitation and/or the fact that benthic samples probably have similar environmental conditions, if they are close to each other (Supplementary Figure S5; Hanson et al., 2012). Recent study of bacteria using HTS in the lakes and reservoirs indicated that rare bacteria showed a similar spatial pattern with the abundant bacterial subcommunity (Liu et al., 2015). These results indicate that similar structuring processes (e.g., dispersal) can influence benthic dominant, rare and conditionally rare subcommunity compositions (Hanson et al., 2012). However, although there were similar distribution patterns of dominant taxa, RT, and CRT, we found that dominant taxa have a weaker distance-decay relationship (*r* = -0.400) than RT and CRT (*r* = -0.579 and *r* = -0.798, respectively, **Table 1**). This suggests that the benthic dominant microeukaryotic community has a higher capacity to dispersal than RT, hence leading to a more cosmopolitan distribution (Liu et al., 2015).

### Relative Importance of Environmental and Spatial Factors Influencing Microeukaryotic Distribution

Our results indicate that both the environmental and spatial factors play significant roles in structuring the microeukaryotic metacommunity, although the relative effects of the two types of influencing factors on community variations were different (**Table 2**). The mechanisms underlying the assembly processes are important question in the field of microbial ecology (Hanson et al., 2012). The different relative effects of environmental and spatial factors influencing MCCs may be ascribed to the dominant taxa, RT, and CRT which had different community compositions and properties (Liu et al., 2017). In aquatic ecosystems, the responses of microbial communities to environmental and spatial changes are mediated by their properties; such as physiological tolerance, dispersal capacity, taxonomic and functional diversity (Gong et al., 2015; Liu et al., 2015). Our result was consistent with previous study on aquatic and sedimentary bacteria communities in 16 lakes from western China (Yang et al., 2016). However, our study was not in agreement with another study in Antarctica coastal lakes (Logares et al., 2013), which suggested that MCC was strongly influenced by environmental factors (e.g., salinity) and weakly correlated with geographical distance. This inconsistency maybe attributed to the spatial distance differences among the studied area and different environmental gradients (e.g., salinity ranged from 0 to 100 psu in coastal lakes in Antarctica vs. 12.6 to 30.9 psu in water habitat and 23.2 to 30.0 psu in sediment habitat in Xiamen) (Logares et al., 2013). In addition, our VPA confirmed that a large proportion of the observed MCCs variations could not be explained, especially for the RT (78.1–97.4% of variance unexplained) and CRT (49.0–81.0%), indicating that more complex mechanisms may generate and maintain the rare biosphere diversity in the coastal waters and intertidal sandy sediments. The unexplained variation may be due to unmeasured environmental and ecological factors, or interactions between taxa and species’ own vital rate. For example, Barberán and Casamayor (2010) found that species sorting solely dominated microbial community composition. Evolutionary drift (stochastic genetic diversification), and ecological drift (stochastic processes of birth, death, colonization, and extinction) could also contribute to spatial effects and unexplained variation (Hanson et al., 2012). Further, another studies indicated that the co-occurrence correlations among microbes should also be responsible for the community structure (Lima-Mendez et al., 2015; Wei et al., 2016). These findings suggested that extending the number of sampling sites, and integrating more environmental factors, along with conducting manipulative experiments across space and time, are necessary for better understanding of the influence and mechanisms of environmental selection and stochastic processes on the biogeography of planktonic and benthic microeukaryotes (Yeh et al., 2015). Therefore, the relative importance of spatial and environmental factors on microeukaryotic distribution awaits further investigation given the unexplained variation.

After removing sites N2 and N3, interestingly, the contribution of environmental factors to the planktonic MCC variations exhibited a rapid decline among all of total, dominant, rare, and conditionally rare planktonic taxa, whereas the contribution of spatial factors did not show significant variation. This result confirmed that environmental factors had a stronger influence on microbial biogeography than spatial factors in these 12 water sites than for the 10 water sites in this study (**Table 2**). More details of sites N2 and N3 were reported in the previous study (Yu et al., 2015).

Finally, we fitted the NM of community assembly for determining whether stochastic processes could explain variation of MCCs (**Figure 4** and Supplementary Figure S6). The NM was very powerful in explaining microbial community structure (*R*^2^ = 0.76) within aquatic environments in a previous study (Roguet et al., 2015). In this study, the fit to the NM showed that stochastic processes appeared to have more influence in total benthic MCC (*R*^2^ = 0.43, 12 sediment sites) compared with total planktonic MCC (*R*^2^ = 0.25, 12 water sites). After removing sites N2 and N3, however, this model explained 72% of the variation in planktonic MCC (*R*^2^ = 0.72, 10 water sites, **Figure 4**). These results indicate that stochastic processes contributed the most impact in planktonic MCC without sites N2 and N3 (Sloan et al., 2006), and this finding may be ascribed to high and random dispersal rate of microeukaryotes in surface water. Their movements are more restricted in sediment than in water habitat, where they can disperse randomly or passively with ocean currents at regional or local scales. The NM findings (the *R*^2^-value of 12 water sites was 0.25 and 12 sediment sites was 0.43, respectively) were almost fully consistent with results revealed by VPA (the community variation explained by spatial factors for plankton and benthos), although the contributions of spatial factors to the MCC variations differed between the two statistical methods. The difference may be attributed to the MCC variation explained by VPA only involve the influence caused by geographical distance of sampling sites (dispersal). However, various stochastic-related processes (drift and other mechanisms) could contribute major influence to the MCC variation in NM (Sloan et al., 2006, 2007).

## Conclusion

Our data demonstrated that MCCs were significantly different between water and sediment habitats, and benthos exhibited significantly higher species richness than plankton largely due to high proportions of RT and CRT. The proportions of OTUs and sequences of Excavata were significantly higher in the benthos than in the plankton, whereas Alveolata and Diatomea had significantly higher richness and abundance in planktonic than benthic communities. Mantel tests revealed that environmental factors (e.g., salinity, TP, and TN) were significantly related to the variation of benthic MCCs. However, the lack of significant relationship between environmental factors and planktonic MCCs might indicate plankton communities were affected by more complex mechanisms (such as biotic interactions, stochastic processes and other unmeasured environmental variables). Further, benthic MCCs (including total, dominant, rare, and conditionally rare taxa) exhibited a significant distance-decay relationship, whereas the planktonic communities did not. However, more than 70% variation in planktonic community was well explained by stochastic processes when removing two unique sites (N2 and N3). Both the environmental and spatial factors play significant roles in structuring total and dominant microeukaryotic metacommunities although their relative effects on the planktonic and benthic community variations were different. However, unlike the higher explained variation of the total and dominant taxa, the high proportion of unexplained variation in the RT and CRT indicated that more complex mechanisms may influence the rare subcommunity assembly in the coastal waters and intertidal sediments. Altogether, our results indicate that patterns and processes in marine microeukaryotic community assembly may differ among the different habitats (coastal water and intertidal sediment) and different MCCs (total, dominant, rare and conditionally rare microeukaryotes).

## Author Contributions

WZ conceived the idea and designed the comparison experiments. YP and LY collected the samples and performed the experiments. WC, JY, and WZ analyzed the data. JY and WZ contributed reagents/materials/analysis tools. WC, JY, and WZ wrote the paper.

## Conflict of Interest Statement

The authors declare that the research was conducted in the absence of any commercial or financial relationships that could be construed as a potential conflict of interest.
