# Supplementary material for: Patterns and Processes in Marine Microeukaryotic Community Biogeography from Xiamen Coastal Waters and Intertidal Sediments, Southeast China
Source: Front Microbiol. 2017 Oct 12;8:1912. doi: 10.3389/fmicb.2017.01912 (PMC5644358; doi:10.3389/fmicb.2017.01912)
Supplement: Supplementary file 1 [file Data_Sheet_1.docx]

**Patterns and Processes in Marine Microeukaryotic Community Biogeography from Coastal Waters and Intertidal Sediments**

Weidong Chen^1,2^, Yongbo Pan^1^, Lingyu Yu^1,2^, Jun Yang^2*^ and Wenjing Zhang^1^*

*^1^ State Key Laboratory of Marine Environmental Science, Marine Biodiversity and Global Change Research Center, College of Ocean and Earth Sciences, Xiamen University, Xiamen, 361102 China, ^2^ Aquatic EcoHealth Group, Key Laboratory of Urban Environment and Health, Institute of Urban Environment, Chinese Academy of Sciences, Xiamen, 361021 China*

* Correspondence:

E-mail: zhangwenjing@xmu.edu.cn (Wenjing Zhang), jyang@iue.ac.cn (Jun Yang)

**Supplementary Material**

**Summary**

The supplementary material includes 6 figures and 4 tables.


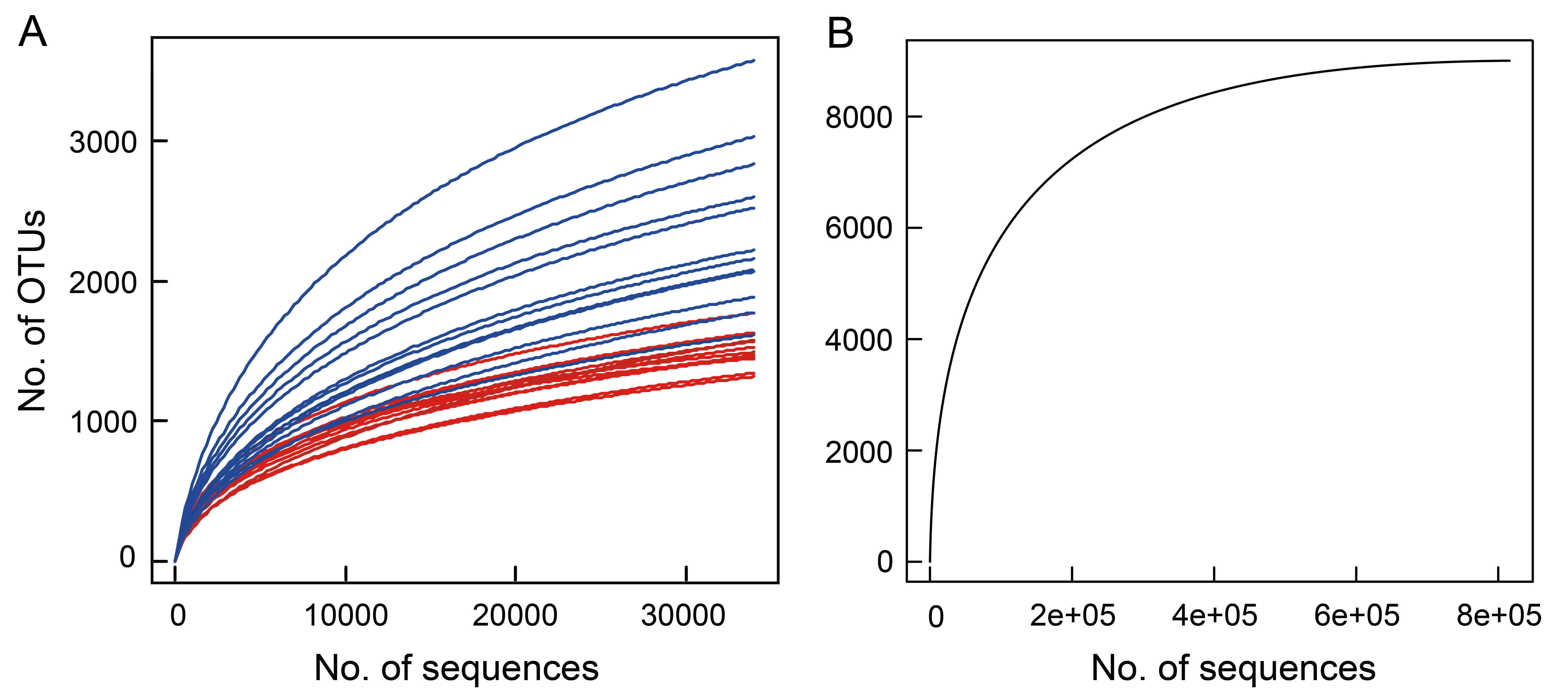


**FIGURE S1 | Rarefaction curves of similarity-based operational taxonomic units (OTUs) at 97% sequence similarity level.** (A) the individual samples, red and blue lines indicate planktonic and benthic communities, respectively; (B) the combined set of 24 samples.

**
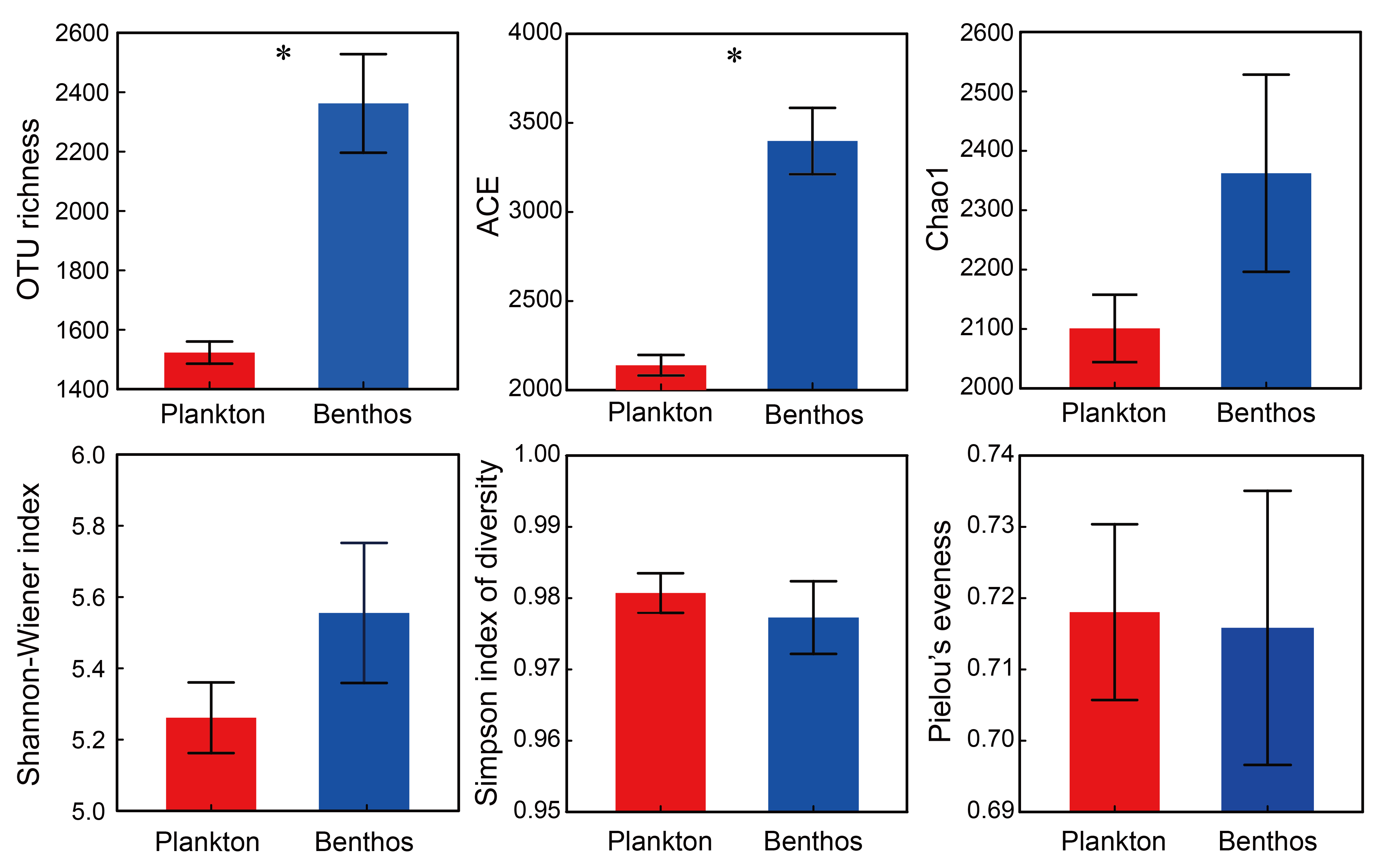
**

**FIGURE S2 | Comparison of** **richness and diversity indices for microeukaryotic communities between water and sediment habitats.** The OTUs were defined at 97% sequence similarity level. All values are means ± s.e. (n = 12); *, *P* < 0.05 (Student’s t-test).


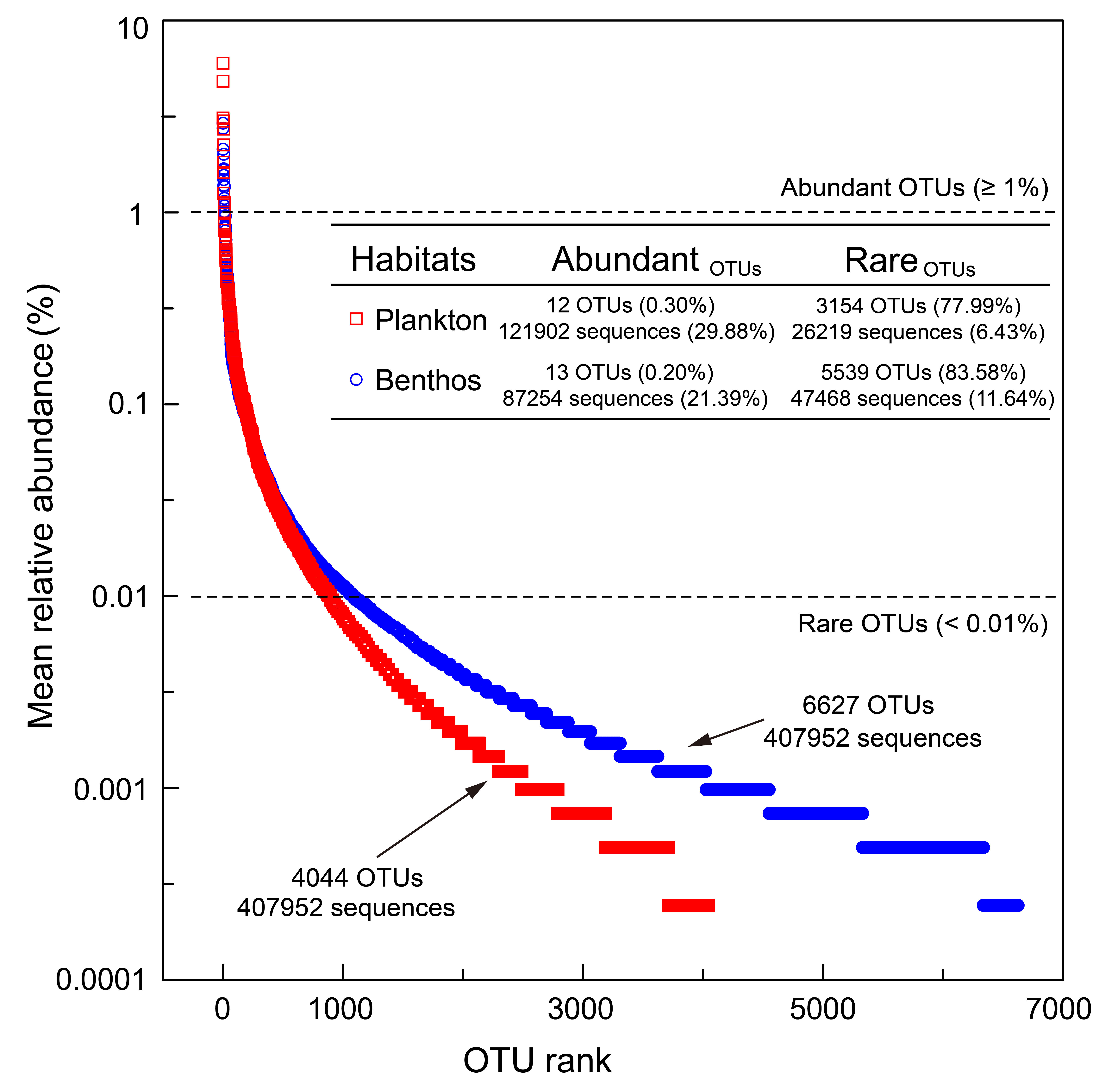


**FIGURE S3 |** **The rank-abundance plots (curves) illustrating the effects of water and sediment habitats in determination of abundant or rare subcommunities.** Dashed lines indicate thresholds for defining abundant (mean relative abundance ≥ 1%) or rare (mean relative abundance < 0.01%) OTUs. The planktonic microeukaryotic metacommunity generated 4044 OTUs from 407952 high-quality 18S rRNA gene sequences, while the benthic microeukaryotic metacommunity generated 6627 OTUs from 407952 sequences. Note that the absolute number of all, abundant and rare OTUs is higher in benthic microeukaryotic metacommunity than planktonic metacommunity. However, a total of 1668 OTUs are common to both planktonic and benthic metacommunities. The relative contributions of OTUs richness or sequence number in plankton and benthos are given in parentheses.


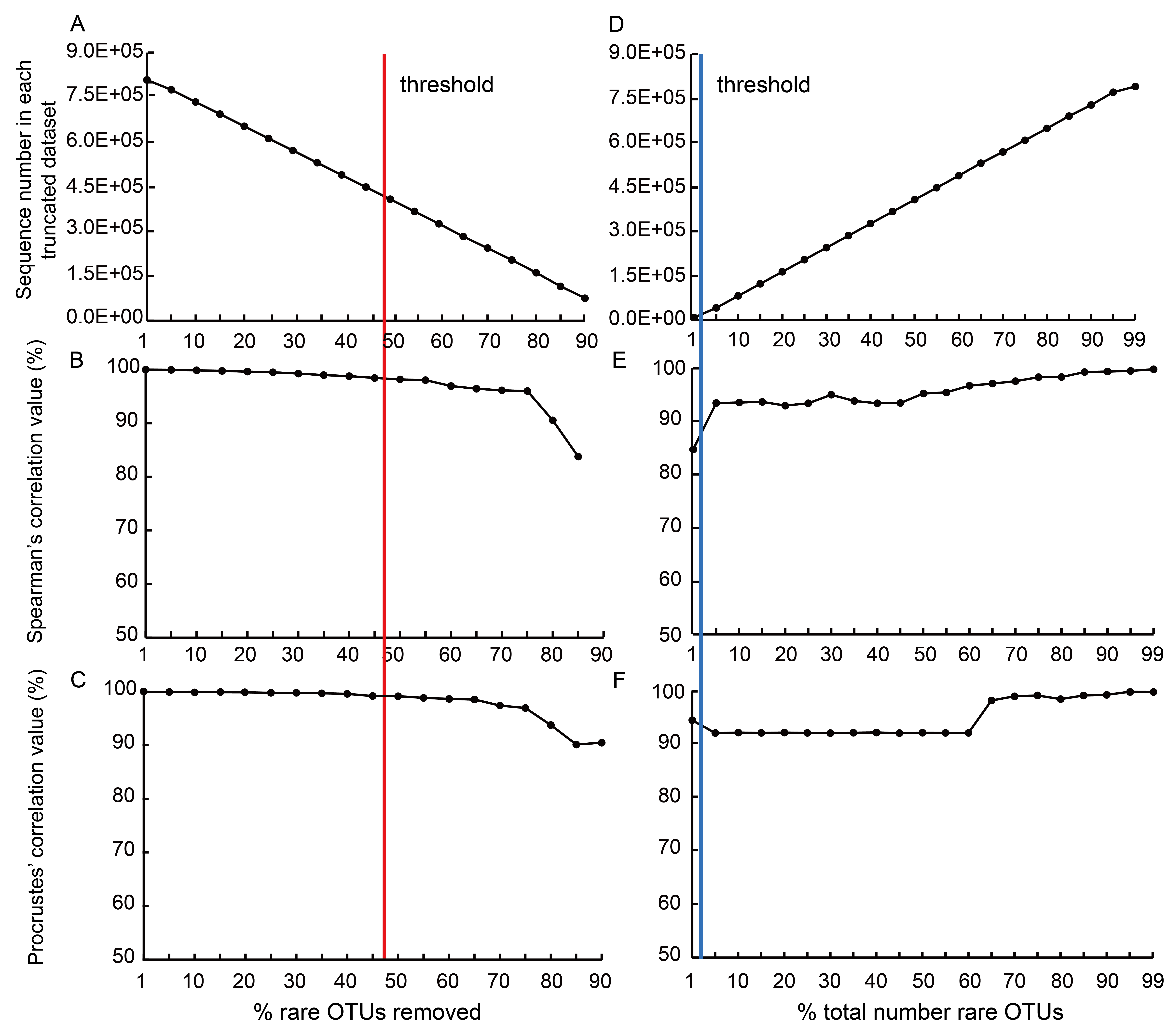


**FIGURE S4 | MultiCoLA profiles based on the dataset-cutoff approaches.** The rare OTUs were removed (A-C), and the rare OTUs were retained (D-F) in each truncated dataset. Abundance of OTUs in each truncated dataset (A, D). Non-parametric Spearman correlations comparing the deviation in complete data structure between the original matrix and truncated matrices (B, E). Comparison of most important axes of extracted variation between the original and truncated datasets (C, F). Lacking points are due to sample loss by applying a given cutoff to the original data set. The red line indicated the threshold of abundant OTUs (47.54%), and the blue line indicated the threshold of rare OTUs (1.88%) in this study, respectively.


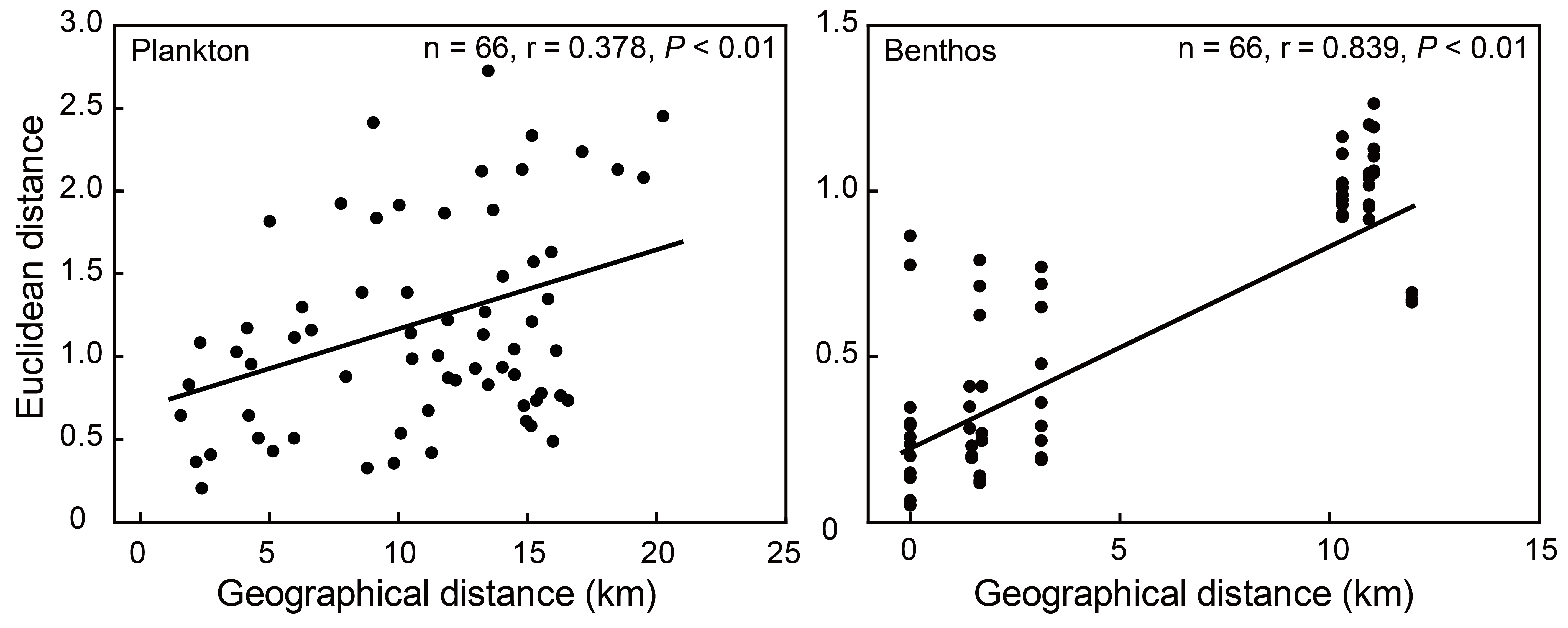


**FIGURE S5 | Correlations between the Euclidean distance of all environmental variables and geographical distance among sampling sites for plankton and benthos.** The n is the number of comparison, and r is the Spearman’s coefficients. For all environmental variables see Table S1.

**
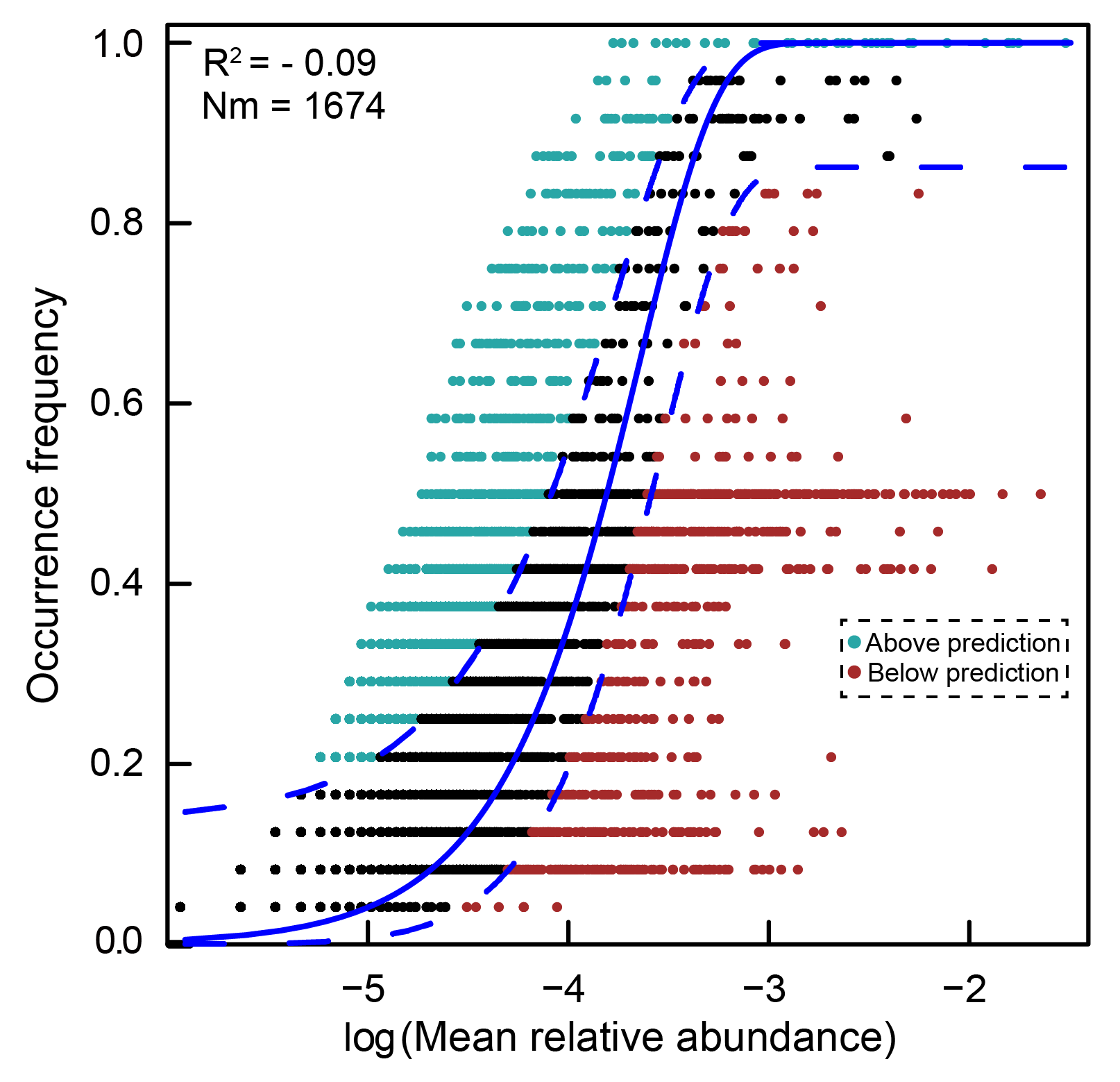
**

**FIGURE S6 | Fit of the neutral model in 24 planktonic and benthic communities.** The solid blue line indicates the best fit to the neutral model and dashed blue lines represent 95% confidence intervals around the model prediction. OTUs that occur more or less frequently than predicted by the neutral model are shown in different colors. *Nm* indicates metacommunity size times immigration, R^2^ indicates the fit to the neutral model. Note that negative R^2^ value indicates no fit to the neutral model.

**Table S1 | Environmental factors for the 12 coastal surface waters and the 12 intertidal sediment pore waters around Xiamen Island.**

| Sample ID | Habitat types | Date | Longitude (E) | Latitude (N) | Temperature (°C) | Salinity (psu) | pH | TN  (mg l-1) | TP  (mg l-1) | DO  (mg l-1) | Turbidity (NTU) | Chl-*a* (μg l-1) | TC  (mg l-1) | NO_X_ -N  (mg l-1) | PO_4_-P  (mg l-1) |
| --- | --- | --- | --- | --- | --- | --- | --- | --- | --- | --- | --- | --- | --- | --- | --- |
|  |  |  |  |  |  |  |  |  |  |  |  |  |  |  |  |
| N1 | Water | 30/07/2013 | 118°04′ | 24°32′ | 30.2 | 25.9 | 9.26 | 0.71 | 0.021 | 6.30 | 9.9 | 3.40 | - | - | - |
| N2 | Water | 30/07/2013 | 118°05′ | 24°33′ | 31.5 | 26.5 | 9.40 | 0.37 | 0.019 | 7.56 | 11.1 | 5.03 | - | - | - |
| N3 | Water | 30/07/2013 | 118°10′ | 24°33′ | 31.7 | 27.4 | 9.80 | 0.57 | 0.025 | 13.06 | 11.6 | 26.85 | - | - | - |
| E1 | Water | 30/07/2013 | 118°12′ | 24°29′ | 28.5 | 30.9 | 9.16 | 0.40 | 0.016 | 5.65 | 21.9 | 2.64 | - | - | - |
| E2 | Water | 30/07/2013 | 118°12′ | 24°28′ | 28.9 | 30.8 | 9.28 | 0.41 | 0.016 | 6.02 | 16.3 | 2.89 | - | - | - |
| E3 | Water | 30/07/2013 | 118°11′ | 24°27′ | 29.2 | 30.5 | 9.31 | 0.33 | 0.014 | 6.21 | 13.5 | 3.18 | - | - | - |
| S1 | Water | 30/07/2013 | 118°10′ | 24°26′ | 28.3 | 30.7 | 9.42 | 0.22 | 0.018 | 6.90 | 12.9 | 9.19 | - | - | - |
| S2 | Water | 30/07/2013 | 118°09′ | 24°26′ | 28.1 | 30.1 | 9.54 | 0.35 | 0.013 | 9.07 | 8.2 | 8.08 | - | - | - |
| S3 | Water | 30/07/2013 | 118°08′ | 24°25′ | 29.3 | 21.3 | 9.29 | 0.83 | 0.019 | 6.27 | 9.3 | 1.88 | - | - | - |
| W1 | Water | 30/07/2013 | 118°01′ | 24°26′ | 29.4 | 12.6 | 8.99 | 1.34 | 0.021 | 5.42 | 20.8 | 4.70 | - | - | - |
| W2 | Water | 30/07/2013 | 118°03′ | 24°28′ | 28.9 | 23.4 | 9.30 | 0.78 | 0.019 | 6.28 | 11.0 | 5.05 | - | - | - |
| W3 | Water | 30/07/2013 | 118°04′ | 24°30′ | 29.2 | 26.6 | 9.42 | 0.49 | 0.019 | 7.78 | 8.3 | 10.41 | - | - | - |
| A1 | Sediment | 02/09/2014 | 118°12′ | 24°30′ | 31.5 | 30.0 | 7.98 | 3.08 | 0.138 | - | - | - | 21.12 | 0.580 | 0.022 |
| A2 | Sediment | 02/09/2014 | 118°12′ | 24°30′ | 31.5 | 30.0 | 8.00 | 2.98 | 0.138 | - | - | - | 18.46 | 0.580 | 0.022 |
| A3 | Sediment | 02/09/2014 | 118°12′ | 24°30′ | 31.5 | 30.0 | 8.01 | 2.99 | 0.138 | - | - | - | 18.85 | 0.580 | 0.022 |
| B1 | Sediment | 02/09/2014 | 118°06′ | 24°26′ | 31.9 | 23.2 | 7.97 | 3.15 | 0.110 | - | - | - | 19.42 | 0.728 | 0.040 |
| B2 | Sediment | 02/09/2014 | 118°06′ | 24°26′ | 31.9 | 23.2 | 7.96 | 3.26 | 0.114 | - | - | - | 16.99 | 0.728 | 0.040 |
| B3 | Sediment | 02/09/2014 | 118°06′ | 24°26′ | 31.9 | 23.2 | 7.98 | 3.27 | 0.114 | - | - | - | 15.87 | 0.728 | 0.040 |
| C1 | Sediment | 02/09/2014 | 118°07′ | 24°26′ | 31.8 | 23.3 | 7.97 | 3.16 | 0.095 | - | - | - | 19.18 | 1.121 | 0.054 |
| C2 | Sediment | 02/09/2014 | 118°07′ | 24°26′ | 31.8 | 23.3 | 7.96 | 2.94 | 0.106 | - | - | - | 16.36 | 1.121 | 0.054 |
| C3 | Sediment | 02/09/2014 | 118°07′ | 24°26′ | 31.8 | 23.3 | 7.98 | 3.10 | 0.102 | - | - | - | 17.96 | 1.121 | 0.054 |
| D1 | Sediment | 1/09/2014 | 118°08′ | 24°26′ | 33.9 | 23.6 | 7.90 | 2.94 | 0.091 | - | - | - | 19.36 | 0.973 | 0.048 |
| D2 | Sediment | 1/09/2014 | 118°08′ | 24°26′ | 33.9 | 23.6 | 7.94 | 2.97 | 0.102 | - | - | - | 16.93 | 0.973 | 0.048 |
| D3 | Sediment | 1/09/2014 | 118°08′ | 24°26′ | 33.9 | 23.6 | 7.96 | 3.01 | 0.098 | - | - | - | 17.33 | 0.973 | 0.047 |

TN: total nitrogen; TP: total phosphorus; DO: dissolved oxygen; Chl-*a*: Chlorophyll*-a*; TC: total carbon; NO_X_-N: nitrate and nitrite nitrogen; PO_4_-P: phosphate phosphorus. - resprents no data available.

**Table S2 | Diversity, predicted richness and Good’s coverage of microeukaryotic community in the 24 samples.**

| Sample ID | Habitat types | OTU richness | ACE | Chao1 | Good’s Coverage |
| --- | --- | --- | --- | --- | --- |
| N1 | Water | 1530 | 2264 | 2256 | 98.34% |
| N2 | Water | 1448 | 1775 | 1749 | 98.92% |
| N3 | Water | 1491 | 1874 | 1868 | 98.80% |
| E1 | Water | 1626 | 2337 | 2261 | 98.31% |
| E2 | Water | 1571 | 2265 | 2243 | 98.34% |
| E3 | Water | 1470 | 2116 | 2063 | 98.46% |
| S1 | Water | 1344 | 2011 | 1937 | 98.52% |
| S2 | Water | 1318 | 1979 | 1939 | 98.55% |
| S3 | Water | 1459 | 2075 | 2052 | 98.49% |
| W1 | Water | 1774 | 2423 | 2400 | 98.32% |
| W2 | Water | 1631 | 2276 | 2219 | 98.39% |
| W3 | Water | 1615 | 2285 | 2232 | 98.36% |
| A1 | Sediment | 2526 | 3647 | 3529 | 97.24% |
| A2 | Sediment | 2599 | 3544 | 3549 | 97.35% |
| A3 | Sediment | 1886 | 2772 | 2699 | 97.90% |
| B1 | Sediment | 1579 | 2579 | 2556 | 97.98% |
| B2 | Sediment | 2835 | 4008 | 3862 | 96.98% |
| B3 | Sediment | 3032 | 4209 | 4051 | 96.86% |
| C1 | Sediment | 1777 | 2767 | 2706 | 97.86% |
| C2 | Sediment | 2162 | 3152 | 3093 | 97.60% |
| C3 | Sediment | 3577 | 4711 | 4595 | 96.62% |
| D1 | Sediment | 2223 | 3175 | 3097 | 97.58% |
| D2 | Sediment | 2082 | 3142 | 3083 | 97.58% |
| D3 | Sediment | 2069 | 3078 | 2963 | 97.63% |
| 24 sites | Water and Sediment | 9003 ± 24 | 9026 ± 47 | 9005 ± 1.74 | 99.99% ± 0.03% |

The operational taxonomic units (OTUs) were defined at 97% sequence similarity threshold. For the combined set of 24 sites’ samples, data are means ± s.e. (n = 24).

**Table S****3 | Contribution of each microbial taxa category to microeukaryotic community in the combined set of 24 samples.**

|  | OTU number | Sequence number |
| --- | --- | --- |
| All OTUs | 9003 | 815904 |
| Abundant taxa (AT) | 0 | 0 |
| Conditionally abundant taxa (CAT) | 8 (0.09%) | 91829 (11.25%) |
| Moderate taxa (MT) | 5 (0.06%) | 7036 (0.86%) |
| Rare taxa (RT) | 3075 (34.16%) | 15360 (1.88%) |
| Conditionally rare taxa (CRT) | 5783 (64.23%) | 365425 (44.79%) |
| Conditionally rare and abundant taxa (CRAT) | 132 (1.47%) | 336254 (41.21%) |

The operational taxonomic units (OTUs) were defined at 97% sequence similarity threshold.

**Table S4 | The most abundant OTUs (mean relative abundance** ≥ **1%) in water and sediment habitat types.**

| Dominance rank | Habitat | OTU ID | Sequence  abundance (%) | Taxonomy |
| --- | --- | --- | --- | --- |
| 1 | Water | OTU_1 | 6.01 | Maxillopoda, Arthropoda, Animalia |
| 2 | Water | OTU_2 | 4.83 | Maxillopoda, Arthropoda, Animalia |
| 3 | Water | OTU_7 | 3.11 | Mediophyceae, Diatomea, Stramenopiles |
| 4 | Water | OTU_5 | 3.01 | Mediophyceae, Diatomea, Stramenopiles |
| 5 | Water | OTU_7452 | 2.72 | Mediophyceae, Diatomea, Stramenopiles |
| 6 | Water | OTU_3 | 2.25 | Maxillopoda, Arthropoda, Animalia |
| 7 | Water | OTU_13 | 1.82 | Maxillopoda, Arthropoda, Animalia |
| 8 | Water | OTU_15 | 1.62 | Mammalia, Animalia |
| 9 | Water | OTU_18 | 1.26 | Mammalia, Animalia |
| 10 | Water | OTU_23 | 1.13 | Mediophyceae, Diatomea, Stramenopiles |
| 11 | Water | OTU_21 | 1.12 | Maxillopoda, Arthropoda, Animalia |
| 12 | Water | OTU_29 | 1.01 | Mediophyceae, Diatomea, Stramenopiles |
| 1 | Sediment | OTU_4 | 2.93 | Mediophyceae, Diatomea, Stramenopiles |
| 2 | Sediment | OTU_6 | 2.75 | Maxillopoda, Arthropoda, Animalia |
| 3 | Sediment | OTU_16 | 2.13 | Maxillopoda, Arthropoda, Animalia |
| 4 | Sediment | OTU_10 | 1.69 | Turbellaria, Platyhelminthes, Animalia |
| 5 | Sediment | OTU_12 | 1.66 | Mediophyceae, Diatomea, Stramenopiles |
| 6 | Sediment | OTU_17 | 1.57 | Nemertea, Animalia |
| 7 | Sediment | OTU_14 | 1.49 | Animalia |
| 8 | Sediment | OTU_22 | 1.38 | Bacillariophyceae, Diatomea, Stramenopiles |
| 9 | Sediment | OTU_11 | 1.36 | Turbellaria, Platyhelminthes, Animalia |
| 10 | Sediment | OTU_26 | 1.18 | Bacillariophyceae, Diatomea, Stramenopiles |
| 11 | Sediment | OTU_31 | 1.15 | Gastrotricha, Animalia |
| 12 | Sediment | OTU_3 | 1.08 | Maxillopoda, Arthropoda, Animalia |
| 13 | Sediment | OTU_7452 | 1.01 | Mediophyceae, Diatomea, Stramenopiles |

The OTUs were defined at 97% sequence similarity level.
